# Genomic variability and population structure of six Colombian cattle breeds

**DOI:** 10.1007/s11250-023-03574-8

**Published:** 2023-05-03

**Authors:** Rodrigo Martinez, Diego Bejarano, Julián Ramírez, Ricardo Ocampo, Nelson Polanco, Juan Esteban Perez, Héctor Guillermo Onofre, Juan Felipe Rocha

**Affiliations:** Corporación Colombiana de Investigación Agropecuaria – Agrosavia, Mosquera, Cundinamarca Colombia

**Keywords:** Admixture, Beadchip, Bovine, Genetic variability, SNP

## Abstract

Analyses of the genetic diversity of indigenous cattle are essential for implementing conservation programs, promoting their sustainable use and maintaining productive advantages offered by these breeds in local conditions. The aim of this study was to investigate the genetic diversity and population structure of six Colombian cattle breeds: Blanco Orejinegro (BON), Costeño con Cuernos (CCC), Romosinuano (ROM), Sanmartinero (SAM), Casanareño (CAS), and Hartón del Valle (HDV). Two additional breed groups were included for comparison: Zebu (CEB) and a crossbreed of Colombian cattle breeds × Zebu. Genetic diversity within breeds was analyzed using expected heterozygosity (He), inbreeding coefficient (f), and runs of homozygosity (ROH). Population structure was assessed using model-based clustering (ADMIXTURE) and principal components analysis (PCA). Zebu cattle showed the lowest genetic diversity (He = 0.240). Breeds with the highest genetic diversity level were HDV and BON (He = 0.350 and 0.340, respectively). Inbreeding was lower for Colombian cattle breeds ranging between 0.005 and 0.045. Overall, the largest average genetic distance was found among the group of Colombian cattle breeds and Zebu, while the smallest was found between ROM and CCC. Model-based clustering revealed some level of admixture among HDV and CAS cattle which is consistent with their recent history. The results of the present study provide a useful insight on the genetic structure of Colombian cattle breeds.

## Introduction

Modern cattle descend from independently domesticated lineages of Taurine and Indicine cattle, which diverged more than 200,000 years ago, with numerous breeds having hybrid ancestry between these different lineages (Murray et al. 2010). Regarding Indicine cattle, Colombia has one of the biggest Zebu populations in the world. These animals have been used in crossbreeding mating systems with Colombian creole cattle, which were originally brought to America by the Spaniards five centuries ago (Martinez et al. 2012). Population structure analysis and breed diversity have provided insight into the origin and evolution of these cattle (Ocampo et al. 2021a, b), and the genetic structure in Colombian breeds such as Blanco Orejinegro (BON) (Martinez et al. 2013) by using low-density panels of microsatellite markers. However, thousands of single nucleotide polymorphisms (SNP) evenly distributed along the genome are currently available to perform genome-wide population analyses in livestock populations (Decker et al. 2014).

SNP genotyping has become an important tool for animal breeding, with different methods available to identify population stratification. These new genotyping techniques offer the possibility to perform thorough assessments of the genetic structure and the relationships between cattle populations. This improves previous population genetic parameter reports like the fixation index (*F*_*ST*_) or inbreeding coefficients, usually estimated from just few genetic markers (Corbin et al. 2012). *F*_*ST*_ is a fixation index that evaluates genetic diversity of animal populations and allows determining the relative genetic distance between these populations (Brito et al. 2017). It is estimated from allele frequencies and their variance, and it has also been used to identify *loci* under selection (Flori et al. 2009). Inbreeding coefficients on the other hand, which have been calculated from pedigree records, are now estimated along with coancestry coefficients by using high-density SNP arrays. Genome-wide estimates of these parameters have higher accuracy as they represent the actual genome percentage that is homozygous (inbreeding) or the actual genome percentage shared by two individuals (coancestry), while pedigree-based estimates are only expectations of such percentages. Moreover, genome-wide estimates can include relationships due to very distant common ancestors, which are not captured by pedigree-based estimates (Cortes et al. 2019).

These population parameters are useful to implement different genome wide evaluation studies (Cortes et al. 2019). Genomic selection causes a larger reduction in inbreeding rates per generation than traditional pedigree best linear unbiased prediction (BLUP) selection; however, it is not inbreeding free. Therefore, measuring and controlling inbreeding is necessary (Ocampo et al. 2019). Consequently, the understanding of the genetic structure across populations is important to achieve genetic progress by using genome-wide association studies and genomic selection strategies (Kijas et al. 2009). Furthermore, genetic diversity and population structure studies among cattle breeds are essential for genetic improvement and for the understanding of their adaptation and the use and conservation of these animal resources (Ocampo et al. 2021a, b).

Considering this, the aim of this study was to investigate the genetic diversity and population structure within and among seven Colombian cattle breeds using the BovineSNP50 BeadChip to explore the inclusion of these cattle populations in future genomic selection programs.

## Materials and methods

### Population

This study included a total of 2182 individuals of Blanco Orejinegro (BON, *n* = 658), Romosinuano (ROM, *n* = 569), Costeño con Cuernos (CCC, *n* = 464), San Martinero (SAM, *n* = 293), Casanareño (CAS, *n* = 50), and Hartón del Valle (HDV, *n* = 34). These animals belong to the Sistema de Bancos de Germoplasma de la Nación para la Alimentación y la Agricultura (SBGNAA; System of National Germplasm Banks for Food and Agriculture of Colombia), which the Colombian Corporation for Agricultural Research (AGROSAVIA) guard and manage. A mating strategy (Nomura and Yonezawa 1996) has been implemented to control inbreeding in these creole cattle populations kept in situ. It involves the splitting of breed groups into several families, with mating males being moved periodically to neighboring families in a circular pattern. Data from the purebred Zebu (CEB, *n* = 34) and the crossbred populations BON × Zebu (*n* = 61) and ROM × Zebu (*n* = 19) were obtained from commercial herds.

Creole cattle populations were located in research centers of AGROSAVIA across the country. There was no need to apply for approval from the Animal Care and Use Committee for the present study since data was extracted from existing herd books and the biological samples had already been obtained for previous projects and kept in an in vitro germplasm bank.

### DNA samples and genotyping

Frozen blood samples from each animal were available from the in vitro bank of SBGNAA. Most of the DNA was obtained from these samples, and in some cases, DNA extraction was carried out from semen straws.

Genomic DNA was obtained with a commercial extraction kit (MoBio Laboratories, Inc., Carlsbad, CA, USA). DNA quantification was made by a spectrophotometric analysis at 260 nm using a NanoDrop ND-2000 spectrophotometer (Thermo Fischer Scientific Inc., USA) and diluted to 50 ng/μL. Subsequently, DNA quality was evaluated using the same equipment to estimate the absorbance ratio at 260 and 280 nm.

Genotyping was performed with the Illumina Bovine SNP50 BeadChip (Illumina, Lincoln, NE), using a HiScan array scanner in the Molecular Genetics Laboratory of AGROSAVIAand following established protocols (Matukumalli et al. 2009). The dataset only included animals with call rates > 98% and SNP with > 95%. SNPs with genotyping errors or with unknown chromosomal positions were excluded after quality control procedures were performed. Likewise, SNPs with call rates < 95%, minor allele frequency < 0.025 or significant departure from the Hardy Weinberg equilibrium within breeds (*P* < 0.01), were also excluded from the analysis. Finally, the analysis included 44496 autosomal SNPs with an average marker density of 56.4 kb.

### Genetic variability and genetic cluster analysis

The expected heterozygosity (He) and inbreeding coefficient (F) were calculated with the genome and het procedures of PLINK v.1.07 software (Purcell et al. 2007). Runs of homozygosity (ROH) were also calculated using PLINK but adjusting the parameters according to published recommendations (Bosse et al. 2012) (–homozyg-density 1000, –homozyg-window-het 1, –homozyg-kb 10, –homozyg-window-snp 20). Cleaning of the dataset and population structure parameters such as inbreeding, heterozygosity, and fixation indexes were determined with PLINK (Purcell et al. 2007). A principal component analysis (PCA) using multi-dimensional scaling (Purcell et al. 2007) was additionally carried out with the same software, and an identity by state (IBS) matrix was constructed.

Furthermore, the ADMIXTURE software was used to implement an unsupervised clustering on the 44,496 autosomal SNPs in all animal datasets. A 100-fold cross-validation was performed to establish the most likely number of clusters (*K*). Then, the *K* value with the lowest cross-validation error was chosen as the best *K* value. A stacked bar plot of these results was obtained with the R© software (http://cran.r-project.org). *F*_*ST*_ values between breeds were used to infer population structure using a neighbor-joining (NJ) clustering method, and the relationship tree was constructed using the MEGA software version 5.0 (Kumar et al. 2018).

## Results

A joint analysis based on all SNP datasets generated for 2181 individuals from seven different populations (BON, CCC, CAS, CEB, ROM, HDV, and SAM) was carried out to evaluate the genetic variability within and among Colombian Creole cattle breeds, and to explore possible mixture with Zebu animals.

The SNP average heterozygosity ranged between 0.310 for SAM and 0.350 for HDV (Table 1), and all breeds showed high heterozygosity values compared to Zebu, which had the lowest values (0.24 ± 0.04). ROH values were lower than *F* values for all breeds except BON. The highest mean number of ROH per individual (*MN*_*ROH*_) and the highest sum of all ROH segments per individual (*S*_*ROH*_) were found in ROM, while the lowest *MN*_*ROH*_ and the lowest *S*_*ROH*_ were observed in HDV.

**Table 1:** Genetic variability in six Colombian creole cattle breeds and an indicine breed

Breed	He^a^	Homozygosity	*F*^b^	ROH^c^	*MN*_*ROH*_^d^	*S*_*ROH*_^e^
BON	0.340 ± 0.03	0.650 ± 0.03	0.056 ± 0.01	0.013 ± 0.01	9.390 ± 4.24	117.780 ± 66.85
ROM	0.320 ± 0.04	0.670 ± 0.04	0.018 ± 0.13	0.013 ± 0.01	15.320 ± 7.03	184.280 ± 96.7
CCC	0.320 ± 0.04	0.670 ± 0.04	0.031 ± 0.12	0.013 ± 0.01	13.190 ± 5.47	163.100 ± 89.52
SM	0.310 ± 0.04	0.680 ± 0.04	0.036 ± 0.12	0.015 ± 0.01	9.670 ± 5.04	111.220 ± 73.06
CAS	0.330 ± 0.02	0.660 ± 0.02	0.045 ± 0.06	0.011 ± 0.01	6.880 ± 5.72	104.210 ± 117.50
HDV	0.350 ± 0.04	0.640 ± 0.04	0.039 ± 0.11	0.013 ± 0.01	6.820 ± 6.05	90.890 ± 87.70
Zebu	0.240 ± 0.04	0.750 ± 0.05	0.155 ± 0.17	0.023 ± 0.02	11.550 ± 6.32	119.020 ± 81.39

^a^Expected heterozygosity

^b^Inbreeding coefficient

^c^Runs of homozygosity

^d^Mean number of ROH per individual

^e^Sum of all ROH segments per individual (Mb)

Data of observed versus expected number of homozygous genotypes was used to estimate the inbreeding coefficient. Its values ranged between 0.018 ± 0.13 for ROM and 0.056 ± 0.01 for BON, with similar values obtained across all the creole breeds. Conversely, a high inbreeding coefficient was observed for Zebu (0.155 ± 0.170) along with the highest homozygosity (0.75 ± 0.05). ROH were low for all creole breeds, with values ranging between 0.011 ± 0.01 for CAS and 0.015 ± 0.01 for SAM. The highest ROH value was obtained for Zebu (0.023 ± 0.015); however, it was considerably lower than the inbreeding value estimated for this breed.

**Fig. 1:**
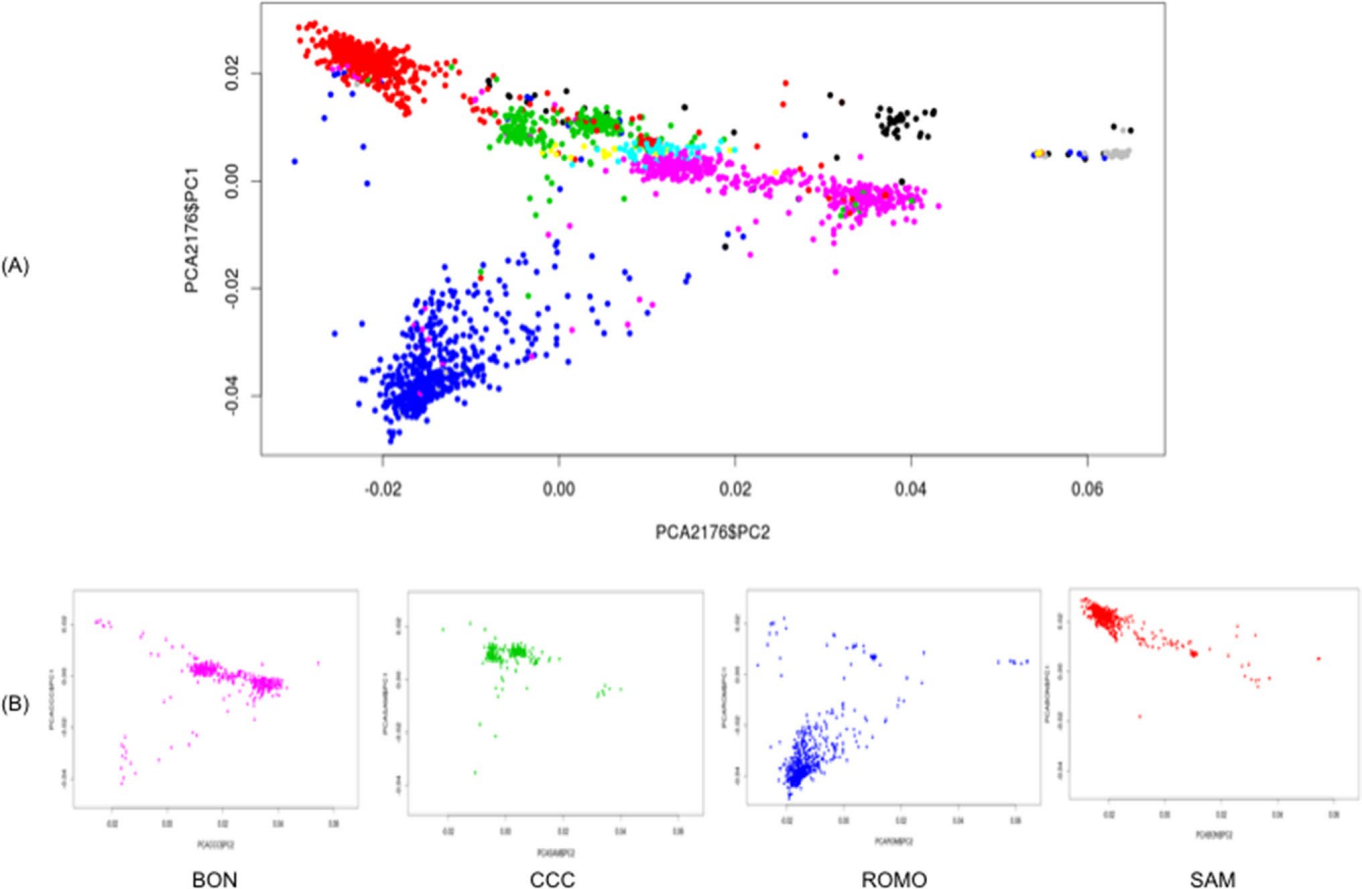
Principal components analysis (PCA) of allele frequency variation in Colombian creole cattle. **A** Principal component analysis in Colombian creole cattle compared to Zebu breed. **B** Principal component analysis for several Colombian cattle breeds. BON, Blanco orejinegro (lilac); CCC, Costeño con cuernos (green); ROMO, Romosinuano (blue); SAM, Sanmartinero (red); CAS, Casanareño (yellow); HDV, Hartón del Valle (cyan), Zebu (grey), crossbred animals (black)

According to the PCA, there was a clear differentiation among groups. The first three principal components explained 14.19% of the variance (6.7%, 4.26%, and 3.23%, respectively). Moreover, creole breeds were separated into four groups, with the BON cattle displaying the highest variation (blue) and located relatively far from the other creole breeds in PC1 (Fig. 1). Furthermore, CAS (yellow) and HDV (cyan) breeds were found close to the centroid in both axes and located between SAM (red) and CCC (green) breeds. On the other hand, Zebu (grey) was found clearly detached from the creole breeds, while the group of crossbred animals (black) included for this analysis was located between Taurine and Indicine breeds on PC2, but clearly differentiated from the pure breeds. Few individuals of CAS (yellow), BON (lilac), and crossbred groups (black) were observed near the Zebu cluster.

**Fig. 2:**
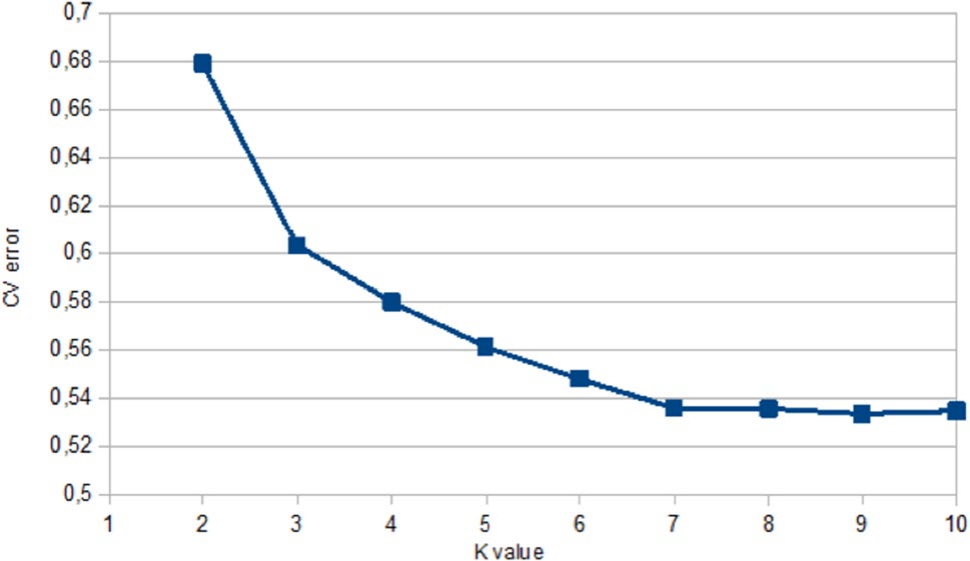
Cross validation error values obtained with the admixture analysis in six creole cattle breeds and Zebu

**Fig. 3:**
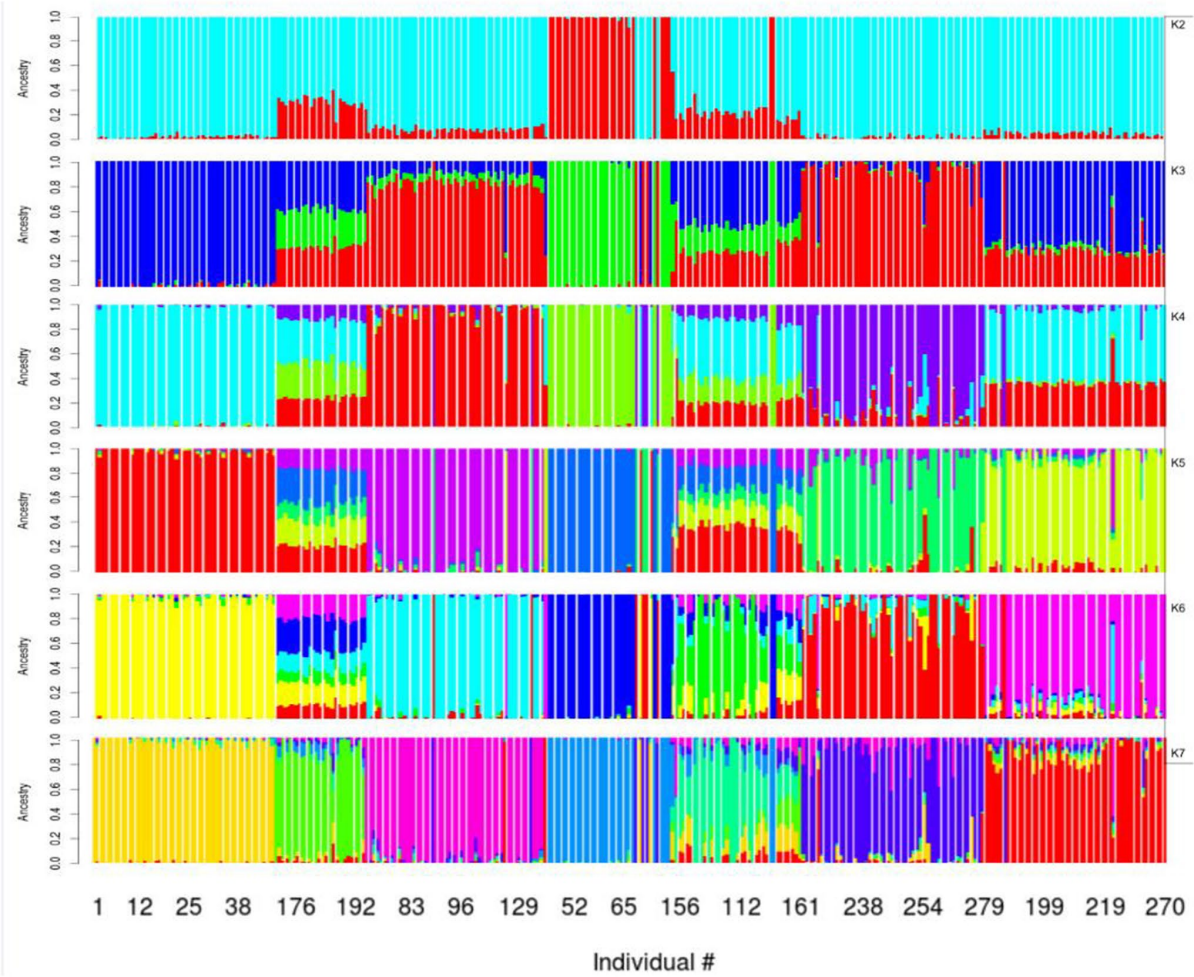
Admixture clustering using six creole cattle breeds and Zebu from *K* = 2 to *K* = 7

Cross validation (CV) values markedly decreased from 0.621 at K = 1 to 0.537 at *K* = 5 with a lower variation found in CV values from *K* = 6 to *K* = 10 (Fig. 2). The optimum number of clusters (*K* = 7) was identified with a CV value of 0.532. When *K* reached values of 4, some creole cattle groups showed differentiation and were represented by well-defined clusters for CCC, ROM, SAM, and BON. The CEB cluster showed a clear differentiation at *K* = 5 and similarly through *K* = 6 and *K* = 7. However, CAS and HDV individuals did not show a strong pure origin, a result that was consistent across all K values (Fig. 3).

**Fig. 4:**
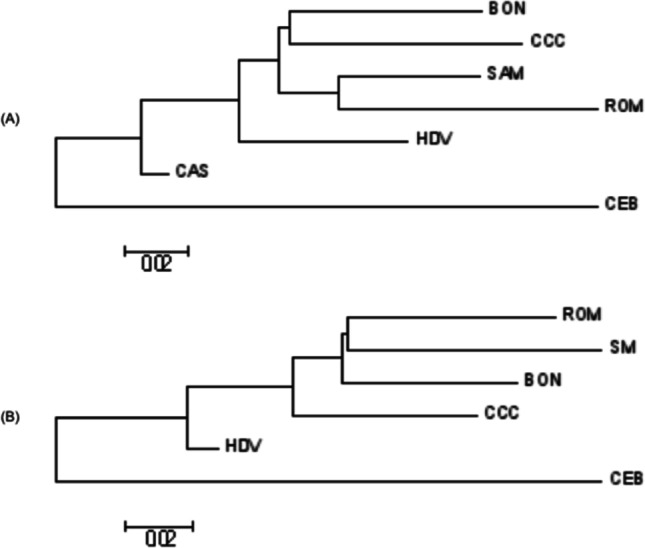
*F*_*ST*_ distances between six Colombian creole cattle and Zebu breeds. BON, Blanco orejinegro; CCC, Costeño con cuernos; ROMO, Romosinuano; SAM, Sanmartinero; CAS, Casanareño; HDV, Hartón del Valle; CEB, Zebu

Additionally, Fst divergences calculated between populations and the neighbor-joining phylogenetic trees are shown in Fig. 4. Phylogenetic trees were constructed for *K* = 4, *K* = 5, *K* = 6, and *K* = 7. In all scenarios, Zebu and Colombian creole cattle breeds were in two different branches. The phylogenetic tree obtained with *K* = 6 and *K* = 7 shows a common node for ROM and CCC, and a common node for CAS and SAM. When *K* = 7, BON is derived from a node that diverges into both the branch that contains HDV and the common node for ROM and CCC.

## Discussion

A whole dataset of SNP genotypes for six different Colombian creole cattle breeds was obtained to assess levels of inbreeding and genetic variability in these populations. Estimated heterozygosity was high for all creole breeds (> 0.31), while the lowest value was observed in Zebu. These genetic diversity levels are higher than those reported by Makina et al. (2016) in indigenous African cattle, with levels of expected heterozygosity that ranged from 0.24 in Afrikaner cattle to 0.30 in the Drakensberg breed. Other breeds included in that study such as Angus and Holstein had the highest levels of gene diversity (He = 0.31). Likewise, McTavish et al. (2013) reported an average heterozygosity of 0.27 in Taurine breeds, while breeds of Indicine origin had an average heterozygosity of 0.16. In that study, the ROM Taurine breed had an average heterozygosity of 0.27. Furthermore, studies of genomic divergence using high-density BeadChips with more than 750,000 SNP (Porto-Neto et al. 2013) also confirmed this trend, with average heterozygosity values of 0.21 and 0.29 for Zebu and Taurine cattle, respectively. This lower genetic diversity observed in Zebu might be due to the effect of SNP ascertainment bias. As many other genotyping arrays, the Illumina Bovine SNP50 BeadChip contains biased sets of pre-ascertained SNP, causing an under representation of fixed genetic differences between taurine and indicine cattle, or leading to passing over sites that are polymorphic only in indicine cattle (McTavish et al. 2015), which in turn would affect the calculation of heterozygosity in these breeds.

A possible explanation of the higher genetic diversity of the Taurine breeds analyzed in this study could be the lower levels of selection pressure applied to the Colombian creole cattle, preventing these populations from undergoing heterozygosity erosion. On the other hand, and as discussed by Ben Jemaa et al. (2018), the bias contained in the Bovine SNP50 BeadChip caused by the inclusion of SNP mostly derived from sequences obtained from European cattle breeds might lead to a biased genetic divergence estimation between more distantly related populations, as the one found between Colombian creole breeds and Zebu cattle. Nevertheless, Gautier et al. (2010) found that hybrid populations had higher polymorphism levels than their population of origin, including hybrids resulting from crosses between European Taurines and Zebu cattle. Thus, the BovineSNP50 would adequately describe the genetic variability of the Colombian creole breeds, which have a Taurine origin, and this would possibly not underestimate polymorphism levels of crosses between these cattle and breeds of Indicine origin.

Inbreeding levels estimated for Colombian creole cattle were low (0.056 ± 0.01 to 0.018 ± 0.). Inbreeding coefficients for these breeds had been calculated using pedigree information (Ocampo et al. 2021a, b; 2020). Furthermore, Martinez et al. (2012) found values between 0.018 for BON and 0.122 for ROM. The lower inbreeding levels found in ROM might be attributed to the low selection pressure these animals have undergone under the conservation program they have been kept for more than 20 years, which includes a circular mating system to control rates of inbreeding (Martinez et al. 2012). Additionally, the effective population size per generation for this breed (Martinez et al. 2012) is consistent with a desired effective population size for conservation of small populations (Meuwissen 2009). Conversely, high inbreeding levels were observed in Zebu (0.155 ± 0.170), which also showed the highest homozygosity value (0.75 ± 0.05). A high inbreeding in Zebu might be caused by its high historic selection pressure for productive traits and by a low effective population size. On the other hand, inbreeding levels can change drastically according to the history of the population analyzed. For instance, the study published by Makina et al. (2016) showed very low levels of inbreeding for Afrikaner (0.004) and for Drakensberger (-0.002) cattle; however, allele frequencies may not be a good inbreeding estimate, although a change over time of this parameter in a particular population might be more useful. Hence, inbreeding levels should be monitored with a regular frequency in Colombian creole breeds, even when they show low inbreeding levels.

Moreover, ROH values were lower than the inbreeding coefficients calculated in Colombian creole cattle using genomic information, and also lower than inbreeding levels previously estimated from pedigree information (Martinez et al. 2012). Conversely, McTavish and Hillis (2015) found much higher values for molecular homozygosity and similarity coefficients compared with inbreeding and coancestry coefficients. Likewise, a study in Lidia cattle breed (Cortés et al. 2019) showed a significantly higher mean ROH-based inbreeding coefficients (*F*_*ROH*_) for 50 k SNP chip data (≈ 0.21–0.26) than the pedigree inbreeding coefficient (*F*_*PED*_) (0.13); this might be explained by the fact that pedigree captures only relatively recent inbreeding since pedigree recording started. Different factors such as the minimum length of ROH, minimum number of SNP involved, or even the density of the beadchip used for genotyping make difficult the comparison of ROH and its statistics (*MN*_*ROH*_ and *S*_*ROH*_) with other studies. Further analysis of ROH along with other statistics for each class of ROH length, the relationship between these statistics, and the coverage and distribution of ROH across chromosomes, is beyond the scope of this paper; however, they must be studied in depth for each Colombian breed to have a better understanding of the inbreeding history of these populations.

Clustering animals based on the genetic PCA analysis showed a clear differentiation between Taurine and Indicine cattle, but also among Colombian creole breeds. Results demonstrated that most of the individuals analyzed are purebred, as the estimated breed proportion assigned to a single animal was greater than 0.9 for a particular breed. However, HDV and CAS breeds showed considerable levels of admixture. Also, Porto-Neto et al. (2013) found well-defined clusters of Taurine (Angus, Charolais, and Holstein) and Indicine (Gir, Guzera, and Nelore) cattle, which was correlated with a lower pair-wise *F*_*ST*_ observed for Zebu breeds in comparison to Taurine breeds. The proportion of variance explained by each component in this study (PC1 = 6.8 and PC2 = 4.3) is similar to that obtained by Rodriguez-Ramilo et al. (2015) (PC1 = 6.1 and PC2 = 3.7), with the first component separating the Zebu Brahman from the Taurine breeds and the second component subdividing in turn the Taurine breeds as in the PCA carried out by Porto-Neto et al. (2013) and the one performed in our study. Likewise, the clusters partly overlapped those of other breeds located nearby, which was particularly true for the CAS and HDV breeds. On the other hand, the crossbred individuals fell between the Taurine and Indicine cattle clusters on the PC2 axis. Similarly, the crossbred cattle included in the study of Zhang et al. (2015) was also placed in the middle of the axis between the parental breeds, i.e., indigenous African cattle and European *Bos Taurus* breeds.

Genetic structure patterns were inferred through admixture analysis ignoring breed membership. Results revealed a well-defined structure for animals from the same breed cluster, while introgression signals were only found in two creole cattle breeds, showing that some animals did not have a pure origin while four creole cattle were Taurine, probably with two different clusters and related with their historical origin. Geographically, the first cluster was related to the north coast of Colombia (CCC and ROM) and the second cluster to herds brought from the oriental savannas (SAM) or from the southern part of the country (BON). Likewise, Ocampo et al. (2021a, b) also found similar results using microsatellite markers in Creole cattle to estimate genetic distances.

As expected, the fixation index *F*_*ST*_ was higher between the Colombian creole cattle and Zebu compared to the value obtained among creole breeds (11–18%), indicating a lower genetic structure differentiation in the latter. Similarly, Campos et al. (2017) observed a *F*_*ST*_ of 12% among locally adapted taurine Brazilian breeds, while much lower values were reported in Zebu breeds (5.33%). A similar genetic differentiation was observed by Weerasinghe et al. (2013) between African and European breeds (15%).

In addition, New World cattle such as Texas Longhorns, Corriente, and ROM breeds have showed evidence of having an admixed ancestry between African hybrid cattle and European cattle. However, the Indicine genomic component found in those breeds might be due to a recent introgression with Indicine cattle in the New World, rather than to an ancient admixture (Edea et al. 2013). In our study, there was no evidence of any admixed ancestry with Indicine breeds for most Colombian breeds. However, HDV and CAS had considerable levels of admixture, including admixed ancestry with Indicine breeds. This might be explained by their particular breeding histories. Unlike HDV and CAS, breeds such as ROM, BON, CCC, and SAM have been subject to breeding programs that aim to maintain their genetic diversity over time.

## Conclusions

The existence of six distinct Colombian Creole cattle populations was demonstrated with the use of a genomic analysis. These populations have high genetic diversity and low inbreeding levels compared with Zebu. However, a regular evaluation of these parameters is recommended as the population structures change over time. Finally, all Colombian creole cattle breeds must be essential and viable objects for conservation, since they are genetically unique and have particular characteristics that include its high capacity to adapt to the difficult conditions of the Colombian tropics, surviving under conditions of low food and water availability, and still showing high fertility and resistance to several pathogens.

## Data Availability

The datasets generated or analyzed during the current study, or both, are available from the corresponding author upon reasonable request.
